# The Effect of Duloxetine on Fusion in Rats Undergoing Posterolateral Spinal Fusion

**DOI:** 10.3390/jcm15052087

**Published:** 2026-03-09

**Authors:** Ozan Güner, Murat Erem, Mert Çiftdemir, Ufuk Usta, Nermin Tunçbilek

**Affiliations:** 1 Department of Orthopedics and Traumatology, Trakya University Faculty of Medicine, Balkan Campus, 22030 Edirne, Türkiye; 2 Department of Pathology, Trakya University Faculty of Medicine, Balkan Campus, 22030 Edirne, Türkiye; 3 Department of Radiology, Trakya University Faculty of Medicine, Balkan Campus, 22030 Edirne, Türkiye

**Keywords:** spinal fusion, duloxetine, neuropathic pain, fracture healing

## Abstract

**Background**: Duloxetine, a serotonin–norepinephrine reuptake inhibitor, is widely used both preoperatively and postoperatively in patients with neuropathic low back pain. This study aimed to determine the impact of duloxetine administration on posterolateral spinal fusion in rats and to evaluate the dose-dependent relationship of this effect. **Methods**: A pre-established rat model for posterolateral spinal fusion was employed, and four equal groups were formed, each undergoing posterolateral spinal fusion surgery. Except for the control group, the other groups received duloxetine postoperatively starting on day 1 at doses of 30 mg/kg/day, 60 mg/kg/day, and 120 mg/kg/day for six weeks. All rats were sacrificed after six weeks. Fusion status was assessed using manual palpation, radiological examination with plain radiography, and histopathological evaluation. **Results**: No significant differences were observed between groups in manual palpation scoring or radiological scoring. Histopathological evaluations of new bone formation also showed no significant differences between groups. The number of inflammatory cells was found to be higher in the control group compared to the low- and moderate-dose duloxetine groups (*p* = 0.012). Neovascularization scores were slightly higher in the control group compared to the duloxetine-treated groups (*p* = 0.048). **Conclusions**: In this experimental rat model of posterolateral spinal fusion, duloxetine administration was associated with reduced inflammatory cell infiltration and mildly decreased neovascularization on histopathological evaluation. However, these histological differences did not translate into measurable differences in fusion outcomes, as assessed by manual palpation, radiological scoring, or new bone formation. Overall, postoperative duloxetine treatment did not demonstrate a detrimental effect on spinal fusion success, suggesting that its use for neuropathic pain management may be biologically applicable with respect to fusion healing in this animal model.

## 1. Introduction

Spinal fusion surgery, first introduced by Hibbs and Albee in 1911 for treating spinal Pott’s disease, has evolved over the past century into the primary treatment for various spinal conditions, including fractures, infections, and degenerative diseases [1,2]. The core goal of the procedure is to relieve neural compression and stabilize the spine. With the rise in life expectancy and advancements in surgical techniques and implant technology, spinal fusion has become increasingly common, making successful outcomes necessary for societal health.

Neuropathic pain, caused by a lesion or functional abnormality of the nervous system, has numerous causes, including diabetic neuropathy, post-herpetic neuralgia, trigeminal neuralgia, chronic postoperative neuropathic pain, and malignancy-associated neuropathic pain [3]. Several conditions, including malignancies, infections, spinal trauma, and spondyloarthritis, can lead to neuropathic lower back pain, which is a prevalent problem that significantly impacts quality of life [4]. Serotonin–norepinephrine reuptake inhibitors (SNRIs), such as duloxetine, are used to treat neuropathic pain, including chronic lower back pain. Although spinal fusion can address the root causes of such pain, the impact of duloxetine on the spinal fusion process has not been well studied. Despite advances in surgical methods, pseudarthrosis and revision surgeries remain major concerns.

Duloxetine has demonstrated analgesic efficacy in chronic and neuropathic pain by enhancing inhibitory descending pain pathways in the central nervous system through increased spinal levels of serotonin and noradrenaline [5]. Preclinical studies indicate that duloxetine restores spinal noradrenergic tone and reduces hyperalgesia in neuropathic models; its antihyperalgesic effects are reversed by blockade of α_2_-adrenergic receptors, underscoring its role in modulating descending inhibition [6]. Beyond neurotransmitter modulation, duloxetine also attenuates neuropathic pain by inhibiting microglial activation through suppression of P2X_4_ receptor function and associated inflammatory signaling in the spinal cord [7]. These multimodal mechanisms provide a robust biochemical basis for the evaluation of duloxetine’s effects on spinal fusion and fracture healing in this study.

Duloxetine may influence bone metabolism through its action on serotonin receptors found in bone cells [8]. While serotonin from the brain promotes bone formation, serotonin from the intestines can inhibit it [9]. Tachi et al. [10] demonstrated that duloxetine enhances osteoblast activation induced by prostaglandin E_1_ through the upregulation of p38 mitogen-activated protein kinase. This amplification leads to increased synthesis of osteoprotegerin and interleukin-6, both of which are critical regulators of bone metabolism. Zhou et al. [11] reported that duloxetine mitigates ovariectomy-induced bone loss in mice by inhibiting osteoclast differentiateon via the MAPK/NFATc1 signaling pathway. Kang et al. [12] reported that the use of SNRIs, including duloxetine, was associated with reduced bone mineral density and an increased risk of fractures, particularly among recent users.

Duloxetine is commonly prescribed in spine surgery both preoperatively and postoperatively for the management of neuropathic and chronic pain [13,14]. Despite its frequent use, the effects of duloxetine on spinal fusion outcomes and bone metabolism still need further investigation [5,7]. Understanding the potential impact of duloxetine on bone healing is essential to ensure both effective pain management and optimal surgical outcomes. Therefore, the primary aim of this study was to investigate whether postoperative duloxetine administration exerts any detrimental effects on spinal fusion integrity and fusion biology in a rat posterolateral fusion model, with particular emphasis on dose-dependent histological, radiological, and manual fusion outcomes.

## 2. Materials and Methods

The study was conducted between January and June 2024 and adhered to the ethical standards of the Trakya University Faculty of Medicine. Ethical approval was obtained from the Ethics Committee of Trakya University Faculty of Medicine Experimental Animals and Research Laboratory with the reference number 2023-12/02. This study was conducted in accordance with international ethical guidelines for animal research, including the principles of the Animal Research Reporting of In Vivo Experiments (ARRIVE) guidelines and approval by the Institutional Animal Care and Use Committee (IACUC).

### 2.1. Study Design

Forty rats were divided into four groups to evaluate the effects of different duloxetine doses on spinal fusion. The control group received no duloxetine after surgery. D30, D60, and D120 were respectively administered at doses of 30 mg/kg/day, 60 mg/kg/day, and 120 mg/kg/day of duloxetine daily for six weeks, starting 24 h after surgery. The chosen dosages (low, medium, and high) were determined based on drug biochemistry, human equivalent dose calculations, and findings from previous animal studies [15,16,17].

At the conclusion of the experiment, collaborations with the departments of pathology and radiology were established for histopathological and radiological analysis.

### 2.2. Animals

A preliminary cadaver study was conducted on two rats to assess and standardize the spinal fusion technique. These cadavers were already available in the laboratory and were not included in the experimental groups, in accordance with ethical principles to reduce the use of live animals. Forty female, 8-week-old Sprague-Dawley rats were obtained for this study. Rats (180–220 g) were randomly assigned to four groups of 10 rats each and housed under controlled conditions (12 h light–dark cycle, 19–20 °C). The rats were housed in pairs to ensure their comfort and to avoid disrupting their social behavior. They were only temporarily separated during drug administration. Cages were cleaned every three days. Food and water were replenished daily. No dietary restrictions were imposed; standard dry pellet feed was provided, while ketogenic feed (D12492) facilitated medication administration.

### 2.3. Surgical Procedure

**Figure 1:**
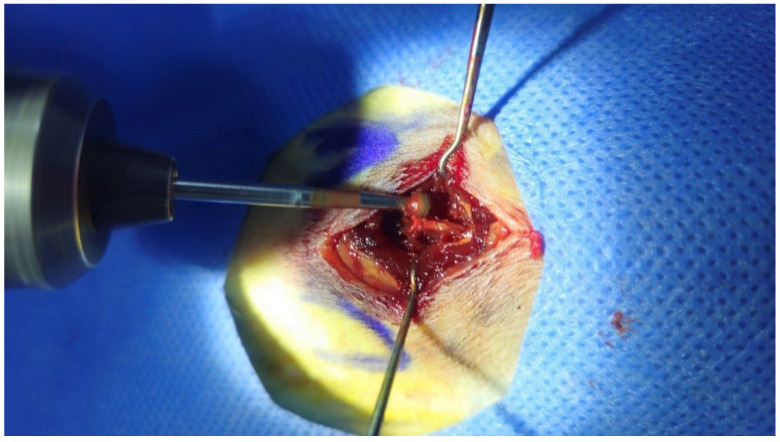
Performing decortication using a burr motor.

On the study’s first day, all rats underwent spinal fusion at the L5–L6 levels under general anesthesia. Anesthesia was induced via intramuscular administration of 10 mg/kg Xylazine Hydrochloride (Rompun^®^, Bayer Healthcare, Leverkusen, Germany) and 90 mg/kg Ketamine Hydrochloride (Ketalar^®^, Pfizer, İstanbul, Turkey). Antibiotic prophylaxis was provided with 10 mg/kg Cefazolin Sodium (Sefazol^®^, Mustafa Nevzat, Ankara, Turkey). Reflexes were assessed to ensure adequate anesthesia depth. The surgical site was shaved, disinfected with 10% povidone-iodine (Poviofix^®^, Naturel Medikal, Ankara, Turkey), and draped. L5–L6 vertebrae and bilateral iliac wings were identified. A 1.5 cm midline incision was made, and skin, subcutaneous tissue, and fascia were dissected. Bilateral facet joints were exposed, their capsules excised, and surfaces decorticated with a high-speed burr to induce bleeding and facilitate fusion (Figure 1). After saline irrigation, paraspinal muscles, fascia, and skin were closed using 3-0 Vicryl^®^ sutures (Ethicon, Bridgewater, NJ, USA). The surgical site was disinfected again, leaving the wound exposed. A single orthopedic surgeon conducted all procedures under veterinary supervision.

### 2.4. Postoperative Period

After surgery, the rats were placed back in their cages and kept warm using heating devices to prevent hypothermia. Respiratory function was closely monitored. Oral feeding began six hours after surgery, and paracetamol was added to their drinking water for analgesia until the 24th postoperative hour [18,19]. No complications were observed, and the wounds healed with epithelialization as the absorbable sutures dissolved, and fur regrew.

The rats’ food intake was monitored daily, and duloxetine was administered in a ketogenic diet at doses of 30 mg/kg/day, 60 mg/kg/day, and 120 mg/kg/day for the treatment groups. The drug, formulated as gastro-resistant micro-pellets with an average diameter of 1 mm, could not be homogeneously dispersed in drinking water. In addition, the pellet size rendered them unsuitable for oral gavage using feeding tubes appropriate for rats weighing approximately 200 g. Therefore, conventional administration methods such as drinking water admixture or direct gavage were not feasible. To maintain the integrity of the gastro-resistant formulation and ensure reliable drug delivery, incorporation into a custom-prepared diet was identified as the most appropriate method.

Throughout the six-week follow-up period, no additional complications were observed. All surgical incisions healed completely. At the end of the study, radiographic imaging was performed under anesthesia (Lorad Selenia Digital Mammography Unit; Hologic, Marlborough, MA, USA). The rats were then sacrificed, and spinal segments were removed for manual palpation and histopathological examination.

### 2.5. Outcome Evaluations

Manual palpation, radiological evaluation, and histological assessments of new bone formation, neovascularization, and inflammatory cell count were performed.

Manual palpation was evaluated using a two-point scoring system. A score of 0 indicated the absence of fusion, whereas a score of 1 represented the presence of fusion [20].

Radiological appearance was graded on a scale from 0 to 4. A score of 0 indicated the absence of bilateral callus formation. A score of 1 corresponded to unilateral non-solid callus formation, while a score of 2 reflected bilateral non-solid callus formation. A score of 3 denoted unilateral solid callus formation, with the contralateral side showing either no callus or non-solid callus formation. A score of 4 represented the presence of bilateral solid callus formation [21].

Histopathological samples were obtained under the supervision of the surgeon by taking sagittal sections from the midline and performing controlled cuts that extended laterally from the midline, with anatomical landmarks, such as the vertebral body and transverse processes, carefully followed to accurately identify the surgical site.

New bone formation was graded semi-quantitatively on a scale from 0 to 3, with 0 indicating no new bone formation, 1 indicating minimal formation, 2 indicating moderate formation, and 3 indicating maximal new bone formation [22].

Neovascularization was evaluated by CD31 immunohistochemical staining and graded semi-quantitatively on a scale from 0 to 3, where 0 indicated no neovascularization, 1 indicated minimal neovascularization, 2 indicated moderate neovascularization, and 3 indicated the maximal degree of neovascularization [23].

Inflammatory cell count at the fusion site was assessed by quantifying the cells observed within the area under hematoxylin and eosin staining at 100× magnification.

All manual, radiological, and histopathological assessments were performed by independent evaluators. The assessors were blinded to group allocation to minimize potential evaluation bias.

### 2.6. Statistical Analysis

Sample size was calculated using G-Power based on the previous literature with an effect size of 0.57, 80% power, and 0.05 significance level, resulting in a total of 40 rats (*n* = 10 per group). Statistical analyses were performed using SPSS software (IBM SPSS Statistics for Windows, Version 20.0. Armonk, NY, USA) at Trakya University Department of Biostatistics and Medical Informatics. Results are presented as mean ± standard deviation and percentages. Normality of quantitative data was tested with the One-Sample Kolmogorov-Smirnov test. Group comparisons for quantitative variables were conducted with the Kruskal-Wallis test, and categorical variables were compared using the Chi-square test. The statistical significance was set at *p* < 0.05. Interobserver reliability was assessed using the intraclass correlation coefficient (ICC) based on a two-way random-effects model with absolute agreement. Effect sizes were calculated using eta squared (η^2^) based on the Kruskal-Wallis test to determine the magnitude of intergroup differences.

## 3. Results

### 3.1. Manual Evaluation

The scores obtained from the manual palpation examination, conducted independently by three researchers, were recorded. The scores of 0.5 or higher were considered indicative of fusion. The interobserver agreement was found to be moderate (ICC = 0.618; *p* < 0.01). Statistical analysis of the manual palpation scores revealed no significant differences between the groups (Table 1).

### 3.2. Radiological Evaluation

**Figure 2:**
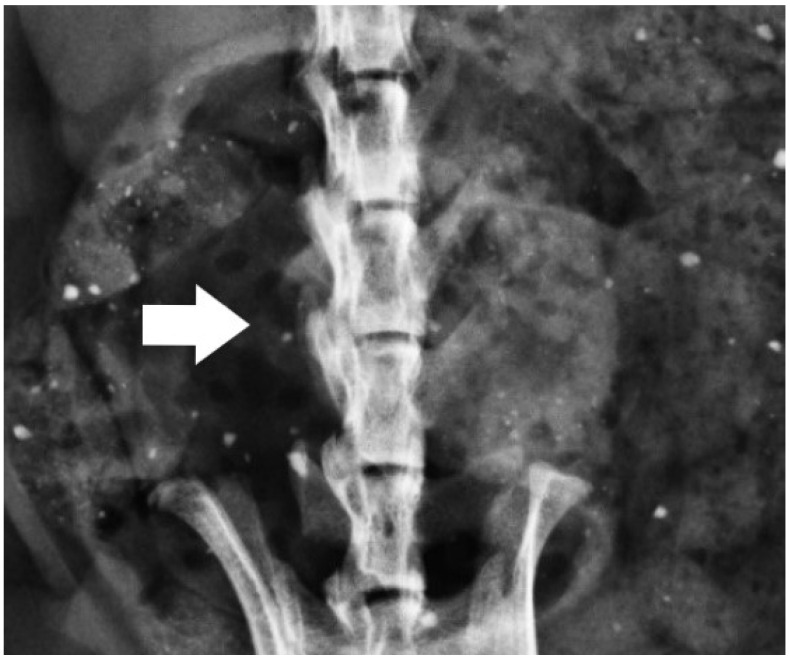
X-ray image of the rat; the arrow shows unilateral fusion. The arrow indicates the fusion area.

In the radiological assessment, three independent researchers recorded the data, and the average scores were calculated. A score of 2.5 or higher was considered indicative of fusion (Figure 2). The agreement among researchers for radiological scoring was also found to be moderate (ICC = 0.523; *p* < 0.01). No statistically significant differences were observed between groups (Table 2).

### 3.3. Histopathological Evaluation

**Figure 3:**
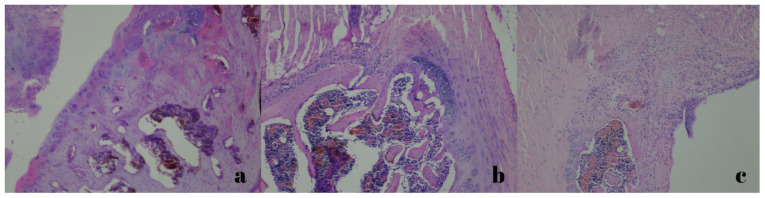
New bone formation shown under ×100 magnification, stained with hematoxylin and eosin. ((**a**): Minimal; (**b**): moderate; (**c**): maximal).

**Figure 4:**
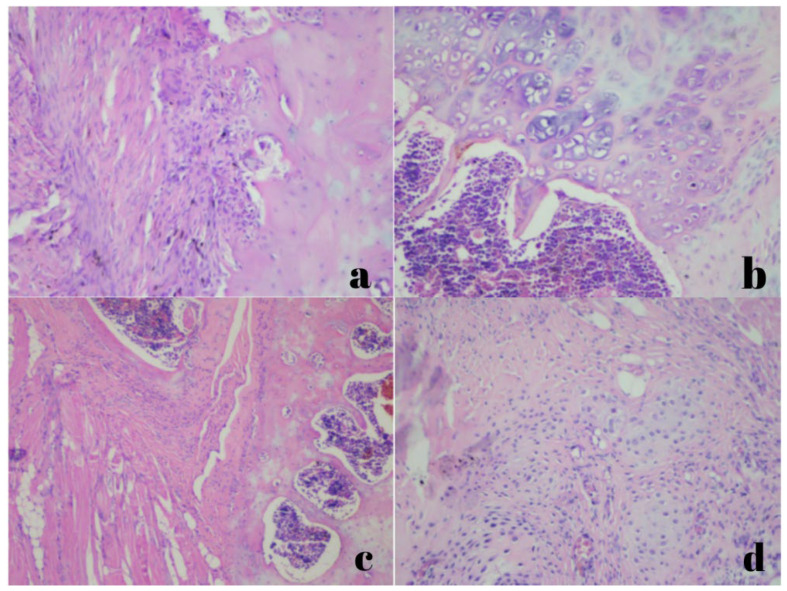
Neovascularization scoring shown under ×100 magnification, stained with hematoxylin and eosin. ((**a**): None; (**b**): minimal; (**c**): moderate; (**d**): maximal).

Histopathological evaluations were conducted by the department of pathology. New bone formation, neovascularization, and inflammatory cell density were assessed (Figure 3 and Figure 4).

A statistically significant difference was observed among the groups in terms of inflammatory cell count (*p* = 0.012). The control group showed a significantly higher count than the D30 group. A similar trend was observed between the control and D60 groups. No statistically significant difference was found between groups in terms of new bone formation. Neovascularization scores were numerically higher in the control group compared to all duloxetine-treated groups (*p* = 0.048); however, the statistical significance of this finding was borderline and should be interpreted with caution. The control group exhibited significantly higher neovascularization scores compared to the D30, D60, and D120 groups (Table 2 and Table 3).

Effect size analysis (η^2^) revealed a large effect for inflammatory cell count (η^2^ = 0.221) and a moderate effect for neovascularization (η^2^ = 0.136). Effect sizes for manual palpation (η^2^ = 0.001), radiological score (η^2^ = 0.028), and new bone formation (η^2^ = 0.022) were small or negligible (Table 4).

A correlation analysis was conducted between the parameters studied. A positive and weakly significant correlation was found between the manual palpation scores and new bone formation scores (r = 0.341, *p* < 0.05). No significant correlation was found between manual palpation and radiological scores, nor between inflammatory cell count and neovascularization scores. No statistically significant difference was observed in the correlation analysis between the radiological score and the inflammatory cell count. No statistically significant difference was observed in the correlation analysis between the new bone formation score and the neovascularization score. However, a positive and strongly significant correlation was found between inflammatory cell count and neovascularization scores (r = 0.708, *p* < 0.01), and a positive and moderately significant correlation was found between new bone formation and neovascularization scores (r = 0.535, *p* < 0.01). No statistically significant correlation was observed between inflammatory cell count and new bone formation scores.

In conclusion, manual palpation scores, radiological evaluation scores, inflammatory cell counts, new bone formation, and neovascularization scores were determined. The results were calculated as mean ± standard deviation (Table 5). Statistical analysis showed moderate inter-rater reliability in both manual palpation (ICC = 0.618; *p* < 0.01) and radiological evaluation (ICC = 0.523; *p* < 0.01).

## 4. Discussion

Duloxetine is commonly prescribed for neuropathic low back pain in humans. However, its potential impact on spinal fusion remains unexplored. This study evaluated its effects using a Sprague-Dawley rat model, widely employed in orthopedic research due to anatomical and physiological similarities to human bone healing mechanisms. The experimental design was informed by previous investigations on duloxetine and spinal fusion. Additionally, female rats were used in the study to reduce hormonal variability and to maintain methodological homogeneity in the evaluation of experimental outcomes [20,24,25].

The selected doses (30, 60, and 120 mg/kg/day) were determined based on previously reported experimental doses in rats, established clinical doses in humans, and consideration of the higher metabolic rate of rodents within a translational framework. Given these interspecies pharmacokinetic differences, relatively higher doses are required in rat models to approximate clinically relevant systemic exposure and enhance translational relevance [17,23,26,27,28,29].

Duloxetine was provided in enteric-coated micro-pellets within hard gelatin capsules, a formulation designed to protect against gastric degradation. However, water dispersion trials revealed adhesion of micro-pellets to container walls. Alternative administration routes, including intraperitoneal and intravenous delivery, were considered but dismissed due to insufficient supporting literature, potential risks of infection and peritonitis, and concerns regarding daily injection-related distress over six weeks [29,30]. Gastric gavage was initially selected to ensure precise dosing but was abandoned due to rat intolerance, stress induction, and risks of aspiration pneumonia.

A ketogenic feed-based delivery method was adopted, enabling effective incorporation of duloxetine micro-pellets. Feed administration occurred during the active phase of the rats’ circadian cycle to optimize bioavailability. To minimize confounding metabolic effects, standard dry pellet feed was provided in the latter half of the active cycle. Housing rats in pairs ensured equitable drug consumption, as confirmed by observation. Concerns regarding differential feed intake were unfounded, as both animals within each cage consumed similar quantities. Physical assessments demonstrated the micro-pellets’ resistance to mastication, preserving their enteric coating integrity until reaching the gastrointestinal tract. The evaluations were performed at the postoperative sixth week, which is a commonly used time point in the literature for this experimental model [18]. While this time point reflects the established phase of tissue healing, earlier assessments could better characterize acute inflammatory responses, whereas later evaluations may provide additional insight into angiogenesis and osteogenic remodeling processes [31].

Manual palpation and radiological assessments revealed no significant differences in spinal fusion rates between control and duloxetine-treated rats across all dosing groups, indicating that duloxetine neither enhances nor impairs fusion. Histopathological analysis demonstrated a significant reduction in inflammatory cell infiltration and neovascularization in duloxetine-administered groups, particularly at low and medium doses. However, these findings were confined to histological parameters and were not corroborated by mechanical or radiological indicators of fusion success. The dissociation between histological findings and fusion outcomes suggests that these histological alterations may not translate into biologically or clinically meaningful impairment of spinal fusion.

Histological quantification in spinal fusion masses is inherently sensitive to sampling location and section orientation, which may introduce variability and potential sampling bias. Therefore, the observed differences in inflammatory cell density and neovascularization should be interpreted cautiously. Furthermore, the borderline statistical significance observed in neovascularization analyses limits the strength of conclusions regarding duloxetine’s anti-angiogenic effects.

No statistically significant differences were observed in manual palpation scoring. Given its subjectivity, manual palpation alone may be insufficient for definitive conclusions regarding fusion efficacy. Radiological assessments also failed to identify significant differences between control and duloxetine-treated groups, supporting the conclusion that duloxetine does not influence fusion.

Histopathological analysis was conducted under the supervision of a pathologist, with tissue sections acquired based on guidance from the spinal fusion surgeon. This method ensured that samples originated from expected fusion sites, minimizing sampling bias. Previous studies have employed fibroblast counting to assess fusion biology [18]. However, fibroblast quantification was not analyzed in this study because it was not possible due to the late stage of healing.

Histological evaluations indicated that, in most samples, the healing process had largely progressed beyond the initial inflammatory phase, although mild residual inflammatory infiltrates were observed in some cases. This pattern is consistent with the advanced regenerative capacity of 8-week-old Sprague-Dawley rats.

Statistical analyses demonstrated intergroup differences in inflammatory cell counts, with lower values observed in duloxetine-treated groups, particularly at lower doses. Furthermore, the combination of statistical significance and a large effect size for inflammatory cell count indicates a robust group effect in this parameter. While no significant differences were detected in new bone formation, neovascularization scores tended to be higher in the control group. However, the borderline statistical significance of neovascularization findings warrants cautious interpretation. The advanced healing stage at the time of evaluation and the high regenerative capacity of the rat model may have attenuated potential differences in osteogenesis, thereby limiting the translational impact of these histological variations.

Although duloxetine appeared to suppress neovascularization, its lack of impact on bone formation and fusion rates suggests limited clinical relevance. Correlation analysis demonstrated a weak but significant positive association between manual palpation scores and new bone formation. However, no correlation was observed between manual palpation scores and radiological findings, inflammatory cell counts, or neovascularization scores. The absence of a strong correlation across scoring modalities further supports the conclusion that duloxetine does not significantly affect spinal fusion. Although the statistical significance for neovascularization was borderline, the moderate effect size suggests that the observed group differences may be biologically meaningful.

Radiological scores, which offer greater objectivity than manual palpation, also showed no correlation with inflammatory cell counts. The increased inflammatory cell presence in the control group, combined with its lack of association with radiological outcomes, suggests that duloxetine mitigates inflammation at lower and intermediate doses. A strong positive correlation was found between inflammatory cell counts and neovascularization scores, suggesting that duloxetine has anti-inflammatory and anti-angiogenic effects at all doses.

This study has several limitations. The study was conducted on an animal model, which may not fully replicate the biological and biomechanical conditions of human spinal fusion. Histological analyses were performed at a single time point: six weeks postoperatively. While this time frame is generally sufficient to assess fusion in young rats, it may not capture earlier or later phase differences in inflammation, angiogenesis, or osteogenesis. Temporal changes in fusion biology and potential delayed effects of duloxetine could therefore not be evaluated. In addition, biochemical and molecular markers of bone metabolism, inflammation, and angiogenesis were not assessed. The lack of cytokine profiling, growth factor analysis, or gene expression studies limits mechanistic insight into the observed histological differences and prevents definitive conclusions regarding the underlying biological pathways affected by duloxetine. The absence of biomechanical testing represents a limitation of this study. Although this approach was chosen to preserve the reliability of histopathological evaluations, it limits the objective assessment of the mechanical strength and functional integrity of the fusion mass. Additionally, drug–receptor interaction analyses and molecular docking may enhance the mechanistic depth and scientific strength of experimental studies. The absence of these analyses in the present study may therefore be considered a methodological limitation. Finally, duloxetine administration was performed using a diet-based delivery method to preserve the integrity of the enteric-coated formulation. Although care was taken to ensure consistent intake, individual variations in consumption cannot be entirely excluded and may have introduced variability in systemic drug exposure.

## 5. Conclusions

This study represents the first experimental evaluation of duloxetine’s effects on spinal fusion. In conclusion, duloxetine administration did not result in significant differences in spinal fusion outcomes as assessed by manual palpation or radiological evaluation. While histopathological analyses revealed reduced inflammatory cell infiltration and relatively decreased neovascularization in duloxetine-treated groups, these findings did not correspond to impaired new bone formation or fusion integrity. Thus, duloxetine appears to modulate certain histological aspects of the healing environment without exerting a detrimental effect on spinal fusion in this rat model. Further research should focus on comprehensive biomechanical and biochemical analyses of duloxetine’s effects in rat models to elucidate its underlying mechanisms and therapeutic potential for neuropathic pain.

## Figures and Tables

**Table 1:** The analysis of manual palpation examination and radiological evaluation between groups.

Variables	Groups				*p* *
	Control *n* (%)	D30 *n* (%)	D60 *n* (%)	D120 *n* (%)	
Manual Palpation Examination					
Fusion exists	5 (50)	8 (80)	5 (50)	4 (40)	0.304
Fusion does not exist	5 (50)	2 (20)	5 (50)	6 (60)	
Radiological Evaluation					
Fusion exists	6 (60)	4 (40)	8 (80)	8 (80)	0.184
Fusion does not exist	4 (40)	6 (60)	2 (20)	2 (20)	

Chi-square test * p < 0.01. D30: 30 mg/kg/day duloxetine-administered group; D60: 60 mg/kg/day duloxetine-administered group; D120: 120 mg/kg/day duloxetine-administered group.

**Table 2:** The analysis of manual palpation, radiological and histopathological evaluations between groups.

	Control *n* = 10 Mean ± SD	D30 *n* = 10 Mean ± SD	D60 *n* = 10 Mean ± SD	D120 *n* = 10 Mean ± SD	*p* *
Manual palpation score	0.53 ± 0.39	0.64 ± 0.29	0.43 ± 0.42	0.40 ± 0.41	0.539
Radiological X-ray score	2.63 ± 0.56	2.36 ± 0.58	2.76 ± 0.74	2.83 ± 0.48	0.262
Inflammatory cell count	9.50 ± 9.19	2.80 ± 4.52	1.00 ± 3.16	4.50 ± 6.12	0.012 *
New bone formation	1.50 ± 0.85	1.90 ± 1.10	1 ± 1.25	1.20 ± 1.14	0.285
Neovascularization	2.00 ± 0.94	1.10 ± 0.88	0.80 ± 1.03	1.00 ± 1.05	0.048 *

Kruskal–Wallis test * p < 0.05. SD: standard deviation. D30: 30 mg/kg/day duloxetine-administered group; D60: 60 mg/kg/day duloxetine-administered group; D120: 120 mg/kg/day duloxetine-administered group.

**Table 3:** Intergroup comparison of manual palpation, radiological scores, inflammatory cell count, new bone formation, and neovascularization scores.

Comparison Groups	Manual Palpation *p*-Value	Radiological Scoring *p*-Value	Inflammatory Cell Count *p*-Value	New Bone Formation *p*-Value	Neovascularization Score *p*-Value
All Groups (Overall)	0.539	0.262	0.012 *	0.285	0.048 *
Control vs. D30	0.553	0.227	0.001 *	0.364	0.001 *
D30 vs. D60	0.278	0.115	0.164	0.092	0.330
D60 vs. D120	0.874	0.816	0.069	0.688	0.629
Control vs. D60	0.558	0.404	0.001 *	0.224	0.001 *
Control vs. D120	0.435	0.604	0.144	0.551	0.001 *
D30 vs. D120	0.178	0.089	0.562	0.172	0.717

Kruskal–Wallis test * p < 0.05. D30: 30 mg/kg/day duloxetine-administered group; D60: 60 mg/kg/day duloxetine-administered group; D120: 120 mg/kg/day duloxetine-administered group.

**Table 4:** Intergroup comparison and effect size analysis.

Comparison Groups	*p* *	Effect Size (η^2^ ‡)
Manual Palpation Scores	0.539	0.001 **
Radiological Scores	0.262	0.028
Inflammatory Cell Count	0.012 *	0.221
New Bone Formation	0.285	0.022
Neovascularization	0.048 *	0.136

Kruskal–Wallis eta-squared test * p < 0.05; ‡ η^2^ magnitudes were interpreted as negligible (<0.01), small (0.01–0.059), moderate (0.06–0.139), and large (≥0.14). ** Negative η^2^ values were interpreted as zero.

**Table 5:** Correlation analysis between manual palpation, radiological scores, inflammatory cell count, new bone formation, and neovascularization scores.

		1	2	3	4	5
1. Manual Palpation Score	r	1.000				
	*p*					
2. Radiological X-ray Score	r	0.253	1.000			
	*p*	0.116				
3. Inflammatory Cell Count	r	0.199	0.131	1.000		
	*p*	0.219	0.419			
4. New Bone Formation Score	r	0.341 *	0.014	0.246	1.000	
	*p*	0.031	0.933	0.127		
5. Neovascularization Score	r	0.268	0.114	0.708 ‡	0.535 ‡	1.000
	*p*	0.094	0.484	0.000	0.000	

Spearman’s test * p < 0.05; ‡ p < 0.01.

## Data Availability

The data presented in this study are available within the article. For further inquiries, additional data can be requested from the corresponding author.
